# Enhanced YOLO-based framework for accurate detection and identification of common wheat impurities with distinct objects

**DOI:** 10.1038/s41598-025-23032-9

**Published:** 2025-11-18

**Authors:** Hossein Bagherpour, Negar Fattahi Peyruo

**Affiliations:** Department of Biosystems Engineering, Faculty of Agriculture, Bu-Ali Sina University, Hamedan, Iran

**Keywords:** Artificial intelligence, Object detection, Wheat impurities, YOLOv5n, Computer science, Software

## Abstract

Real-time detecting and identifying impurities in wheat grain mass is crucial for wheat storage silos, flour mills and modern combines. Depending on the detection objectives, accuracy is typically prioritized in laboratory-based applications, whereas real-time detection scenarios require a trade-off between accuracy and speed. Consequently, detection algorithms should be developed in alignment with the specific performance demands of each application. Given the strong object detection capabilities of YOLO models, four updated algorithms—YOLOv5n, YOLOv5x, YOLOv8n, and YOLOv8x—were employed to achieve the research objectives. In this study, a total of 700 labeled images of three different resolutions, encompassing 11 distinct classes, were used to train the algorithms. The results of this study demonstrated that increasing the model size had no significant effect on mAP but substantially reduced processing speed. For laboratory applications, YOLOv5x and YOLOv8x exhibited nearly identical performance, making them suitable candidates. Among the tested models and image sizes, YOLOv5n with an image resolution of 320 × 320 maintained accuracies while improving detection speed by 4%, making it a suitable choice for real-time applications. Overall, the mAP@50 for impurities with similar visual characteristics, such as wheat grains, sun pest-damaged grains, and shriveled grains, was 88%, 86%, and 85%, respectively, while for other impurities, it exceeded 95%. These findings underscore the potential of YOLO models for impurity detection in wheat, providing a non-destructive testing method that could be extended to impurity recognition in other grains.

## Introduction

Wheat is the most adaptable cereal crop, capable of growing under a wide range of climatic conditions. According to the latest FAO statistics, the global wheat cultivation area spans approximately 220 million hectares, with an estimated production of around 800 million tonnes^1^. The presence of impurities, particularly weed seeds, in wheat grains significantly reduces crop quality. Moreover, certain weed seeds contain toxic and harmful chemical compounds that can cause poisoning and impart an unpleasant taste to bread if contaminated wheat flour is used^2^. Since wheat price and quality are largely determined by the percentage of its impurities, including broken, shriveled grains, grains of other cereals, straw, rye grains, non-cereal seeds, weed seeds, stones, soil, sun pest damaged seed, and etc., accurate detection of these impurities is crucial for storage silos, cleaning centers, and flour mills^3^.

Field investigations have shown that in several countries, assessing wheat impurities is a mandatory requirement when delivering grain to storage silos. Due to the relatively small size of agricultural fields and their scattered distribution across different regions, impurity levels vary considerably from one truckload to another, depending on the individual farmer and harvest conditions^4,5^. Consequently, impurities must be evaluated separately for each delivery batch. Manual inspection, however, is prone to subjectivity, human error, and operator fatigue, and cannot efficiently handle the large volume of samples that must be processed during peak harvest periods. This limitation often results in inconsistent impurity assessments and recurring dissatisfaction among farmers^6^.

To address these challenges, researchers in precision agriculture have increasingly turned to automated assessment methods. Early studies focused on classical machine vision and image processing algorithms, which offered promising results for impurity detection but required complex feature engineering and skilled operators for reliable implementation^7^. More recently, deep learning methods—particularly convolutional neural networks (CNNs)—have gained traction in agricultural applications due to their ability to automatically extract hierarchical features, capture subtle visual differences, and generalize across diverse field conditions. In the context of wheat impurity detection, CNN-based approaches have shown clear advantages over traditional techniques by improving accuracy, reducing dependence on manual expertise, and enabling real-time, high-throughput inspection^8,9^.

Numerous studies have explored seed classification using classical algorithms and machine learning techniques. However, the majority of these studies have focused on identifying different seed varieties or performing qualitative classification^10–13^. For example, the classification of wheat grains into healthy and moldy categories was examined using seven color features and sixteen texture features. Among four artificial intelligence models—SVM, MLP, KNN, and NB—the SVM model exhibited the highest performance, achieving an accuracy of 93%^14^. Similarly, Dubey et al.^15^ employed 45 morphological features and an artificial neural network (ANN) model to classify three wheat species, obtaining classification accuracies ranging from 84 to 94% for each species. In another study focused on classifying wheat varieties, using dense SIFT features with an SVM classifier, the study achieved a wheat grain classification accuracy of 88.33%^16^.

Studies on convolutional neural networks (CNNs) have demonstrated the effectiveness of pre-trained deep learning architectures in seed classification tasks. For example, the performance of InceptionV3, ResNet50, Xception, and InceptionResNetV2 was evaluated for classifying 15 different seed species, achieving an accuracy exceeding 98%^17^. In the classification of 14 rice seed varieties using deep learning models such as InceptionResNetV2, InceptionV3, Xception, VGG19, and VGG16, the results indicated that SVM and InceptionResNetV2 outperformed other classification algorithms^18^. YOLOv5 was applied to classify seeds and assess their quality in mixed-crop planting patterns, with an overall model accuracy of 99%^19^. In studies for detection of weeds in wheat samples, the Scale-Invariant Feature Transform (SIFT) algorithm was conducted to identify three types of weed seeds mixed with wheat grains, achieving a recognition rate of 93.3%^20^. Furthermore, using 12 features and the Naive Bayes (NB) model, the identification of several weed species within wheat was investigated, yielding an accuracy of 96%^21^.

Although numerous studies have evaluated wheat grains using conventional algorithms and artificial intelligence, most have focused on identifying different wheat varieties^22–25^. Only a limited number of investigations have specifically addressed the detection of impurities and weeds. Shen et al.^3^ utilized terahertz spectral imaging combined with a convolutional neural network (CNN) to identify four types of impurities as well as the Ladybug insect. Chen et al.^4^ employed the DeepLabV3 + algorithm to measure wheat residues, including straw and stalks, during the harvesting process. Kaya and Saritas^26^ extracted color and dimensional features of wheat grains and foreign materials, such as straw and chaff, to first remove the foreign materials and then assess wheat quality based on vitreousness. In their study, an artificial neural network (ANN) algorithm was used for classification. In a study, Qi et al.^27^ employed the DeepLab-EDA semantic segmentation model to detect and identify broken grains and two types of impurities—straw and husk—present in wheat during the harvesting process.

A review of the literature shows that most studies on wheat impurity detection have focused on a limited number of categories, with none addressing more than five types^3^. In practice, however, wheat often contains over ten distinct impurity types^5^. This gap limits the applicability and reliability of existing approaches under real-world conditions. Therefore, expanding detection to a broader range of impurities is essential to enhance accuracy and robustness in automatic wheat impurity detection systems. Moreover, bulk analysis methods do not allow for the clear visualization of all impurities and tend to increase detection errors; therefore, sample distribution on a flat surface is necessary to ensure complete visibility and accurate identification^28^. In addition, while real-time applications such as combine harvester monitoring systems prioritize speed^29^, laboratory applications such as impurity assessment in storage silos demand higher accuracy. These considerations highlight the need for algorithms capable of simultaneously addressing a wide impurity spectrum, reducing errors through single-seed analysis, and balancing accuracy with processing speed.

Therefore, the objectives of this study are threefold: (i) to detect a broad spectrum of wheat impurities, thereby extending beyond the limited categories examined in previous research; (ii) to conduct impurity identification at the single-seed level in order to improve detection precision; and (iii) to investigate the application of YOLO architectures for wheat impurity detection, with particular emphasis on evaluating and optimizing the established YOLOv8 and YOLOv5 models to develop an optimal framework for accurate and efficient impurity detection.

## Materials and methods

### Preparing foreign impurities

**Fig. 1:**
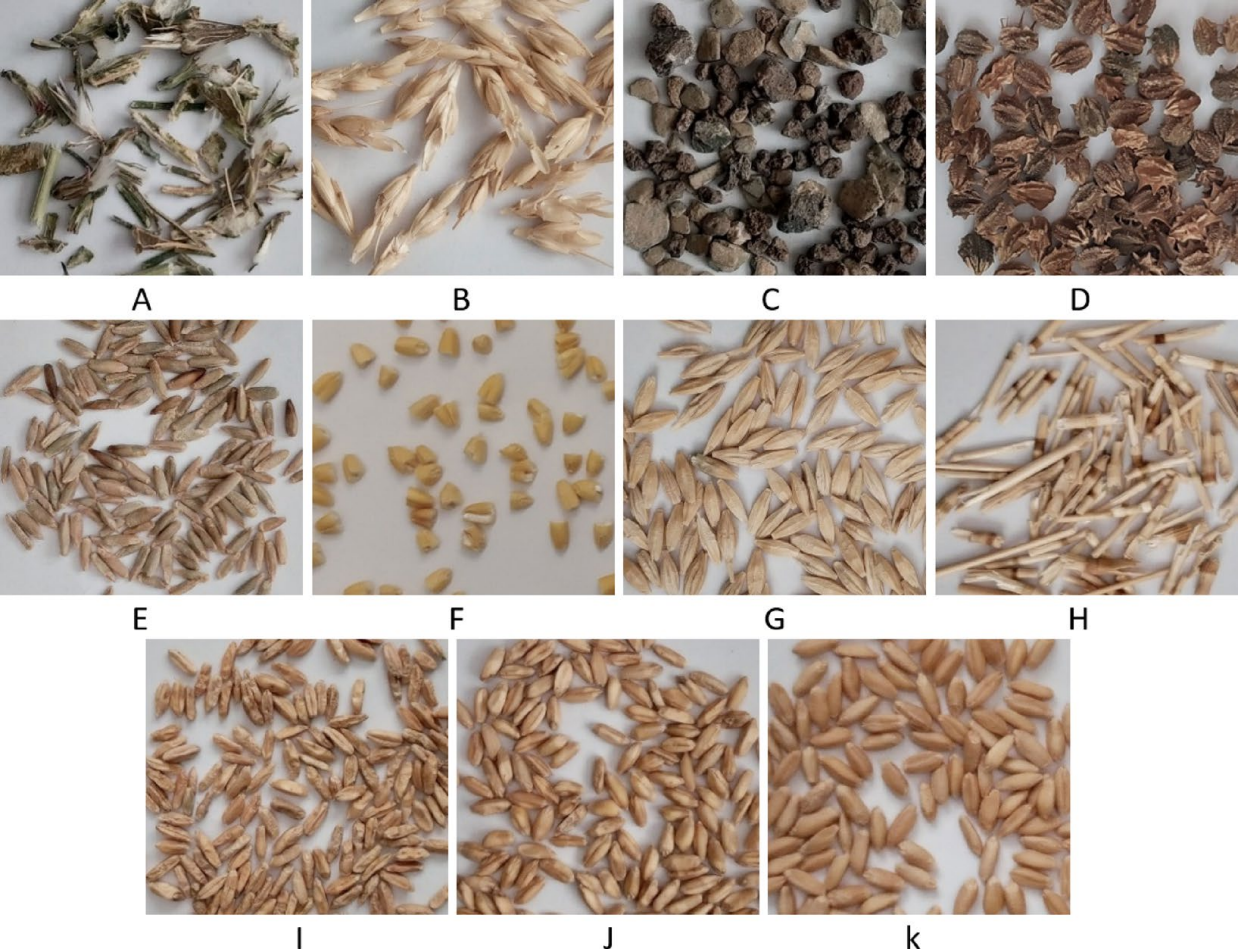
Wheat foreign impurities, (**A**) green weed leaves (weed 2), (**B**) chaff, (**C**) stones and clods, (**D**) cocklebur or weed seeds (weed 1), (**E**) rye seeds, (**F**) broken wheat grains, (**G**) barley grains, (**H**) wheat stems, (**I**) shriveled wheat grains, (**J**), sun pest damaged grains, and (**K**) sound wheat seed.

To enable precise examination and enhance the generalization of the algorithm for identifying various types of impurities, wheat samples were collected from eight storage silos across the provinces of Arak and Hamedan in Iran. These silos are strategically distributed across different locations within the provinces, ensuring a diverse representation of wheat impurities. The collected samples contained 11 distinct types of impurities: cocklebur or weed seeds (weed 1), stones and clods, green weed leaves (weed 2), chaff, wheat stems, barley grains, rye seeds, broken wheat grains, sun pest damaged wheat grains, and shriveled wheat grains. Images of these impurities, presented individually, are shown in Fig. 1, and the number of samples in each class is summarized in Table 1.

**Table 1:** Number of samples in each class and their distribution.

Class	Number of samples	Percentage of total samples (%)
Weed 2	1012	7.15
Chaff	1349	9.53
Stone	628	4.44
Weed 1	1387	9.80
Rye	1044	7.37
Broken	993	7.01
Barley	1271	8.98
Wheat stems	1150	8.12
Shriveled grains	1649	11.65
Sun pest grains	1792	12.66
Sound grains	1884	13.31
Total	14,159	100

### Image acquisition

**Fig. 2:**
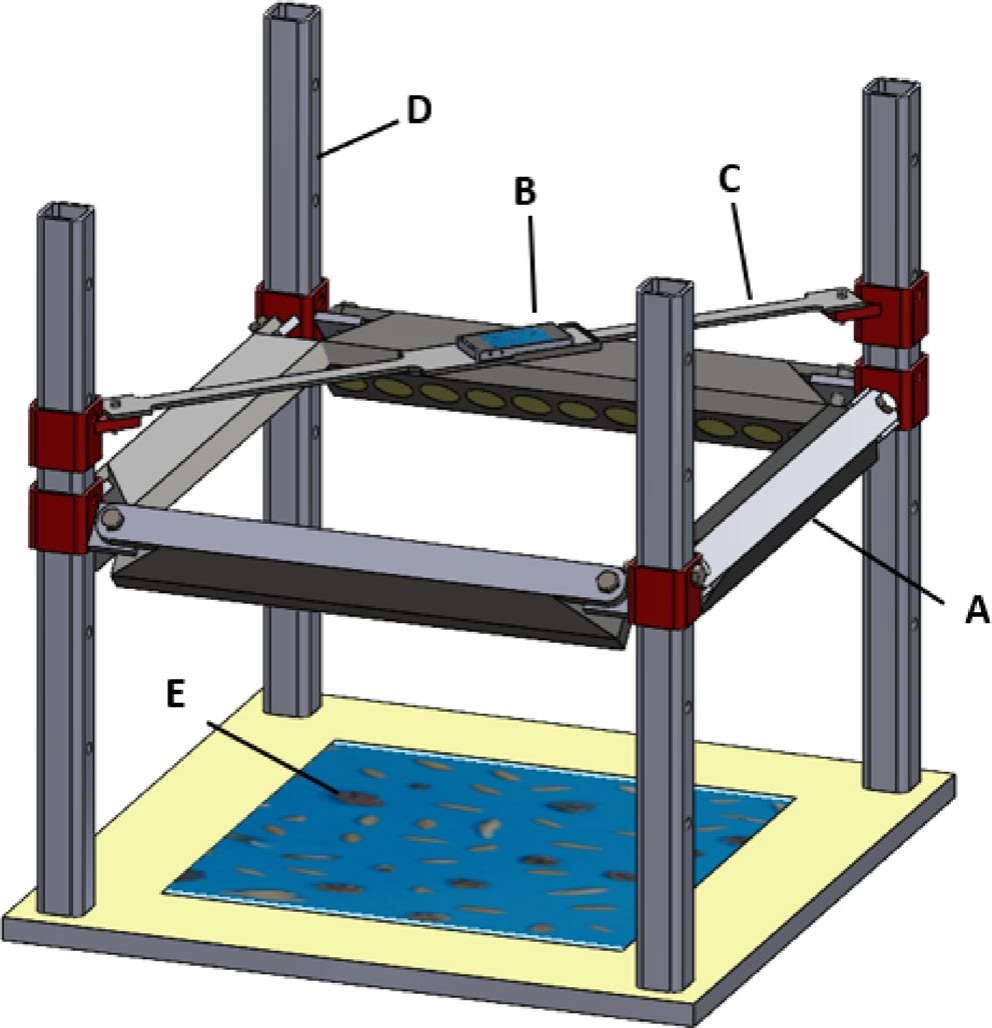
Imaging box, (**A**) Adjustable light source (LED), (**B**) Camera (smart phone), (**C**) Adjustable camera holder, (**D**) Sliding rail, and (**E**) Wheat sample.

Images were captured using mobile camera (Samsung, Galaxy Note 9, pixel size 1.0–1.4 μm and aperture size 1.5–2.4) and saved in JPG format, with a display resolution of 2448 × 2448 pixels. The illumination light source is a 4-segment strip LED diffuse light source (GOLNOR-SMD-30 W), and the light source controller is MD220V1C10A. The light source was installed on an adjustable bracket, which could adjust the distance between the light source and seed sample to provide high brightness and balanced lighting for the system. The mobile camera was placed on the camera holder above the seed and the light source. For capturing images, the wheat sample was manually placed on the blue paper at the bottom of the table. In this study, a total of 700 images were captured. Since each image contained approximately 20 objects, over 14,000 individual objects were analyzed for training and evaluation. Of the total image dataset, 70% was contributed to the training data, 20% to validation data, and 10% to test data. In Fig. 2, a schematic representation of the imaging system is presented.

### Labeling objects

**Fig. 3:**
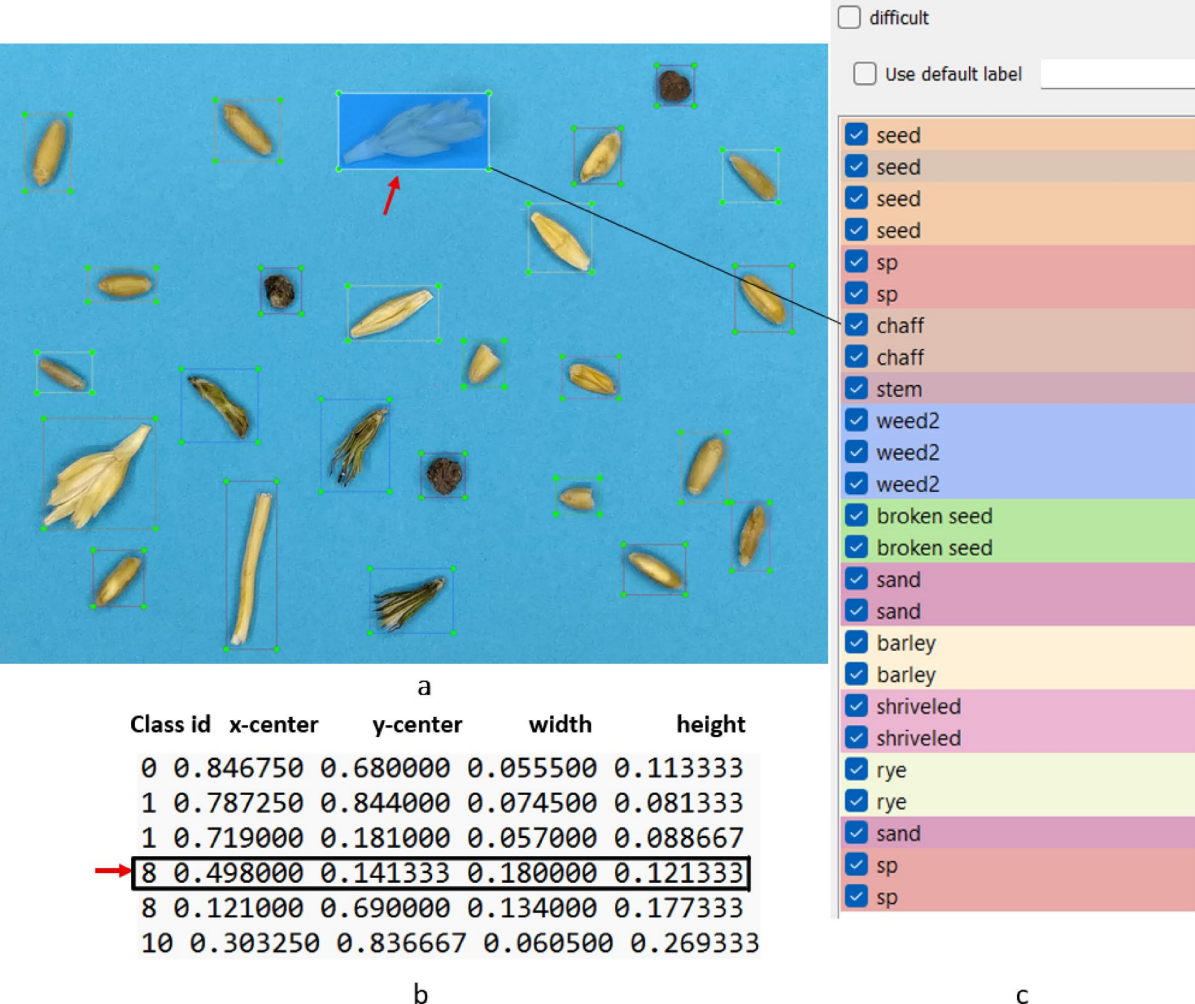
Interface view of the LabelImg application: (**a**) objects, (**b**) object labels, and (**c**) bounding box coordinates (Red arrow shows the bounding box coordinate of the chaff).

For labeling objects within the images, the LabelImg v1.8.0 software was employed^30^. After opening each image in the LabelImg software, the location of all objects was marked with a rectangular bounding box. These bounding boxes are defined by specifying the center coordinates of the rectangle (x_center, y_center), along with its width (width) and height (height). All these values are normalized relative to the dimensions of the image^31^. Figure 3 shows a sample labeled image along with the interface view of the application.

### YOLO models

The You Only Look Once (YOLO), developed by Ultralytics, is a deep learning-based object detection algorithm known for its real-time processing capabilities and high detection accuracy^32^. Unlike traditional object detection methods, which rely on region proposal networks or sliding window approaches, YOLO employs a single neural network to simultaneously predict bounding boxes and class probabilities in a single forward pass. This architecture makes YOLO significantly faster than conventional two-stage detectors while maintaining competitive accuracy. Compared to Faster R-CNN, the new version of yolo model, such as YOLOv8 achieves higher inference speed while maintaining similar accuracy levels, making it more suitable for real-time applications. Compared to SSD, YOLOv8 generally provides better precision due to its refined feature extraction and bounding box regression mechanisms^33,34^.

In this study, we employed two well-known object detection models, YOLOv5 and YOLOv8, and conducted a comprehensive evaluation of their performance. YOLOv5 is recognized for its stability, maturity, and ease of deployment—benefiting from a robust ecosystem, simple API, and efficient training and inference pipelines—making it a popular choice for real-world and edge-based applications^35^. Meanwhile, YOLOv8, the latest iteration from the same developer, introduces architectural innovations such as an improved backbone and neck structures, leading to higher detection accuracy and faster convergence during training^32^. For accuracy-critical scenarios, we used YOLOv5x and YOLOv8x, while YOLOv5n and YOLOv8n were chosen for real-time applications that prioritize speed. This comparison offers a comprehensive evaluation, enabling an informed selection of models best suited to the specific requirements of the study.

The network training parameters are configured with a learning rate of 0.0001, a batch size of 16, and a total of 200 iterations. Transfer learning is employed to reduce model training time. Accordingly, the pre-trained weight file obtained from training the YOLOv8 model on the COCO 2017 dataset is utilized as the initial weight file for training on the wheat seed dataset. This approach facilitates faster network convergence and enhances overall training performance. Moreover, given that the resolution of input images significantly influences the speed and accuracy of deep learning models^36,37^, this study also examines the impact of image size at two resolutions: 640 × 640 and 320 × 320.

### Evaluation metrics

The detection of wheat grains necessitates consideration of both accuracy and processing speed. Therefore, this study employs precision, recall, and mean Average Precision (mAP) as key metrics to evaluate model performance. Additionally, the computational efficiency of the models is assessed using inference time (Per Second).

Precision represents the proportion of correctly classified positive cases within the total predicted positive cases. Precision is determined using Formula (1).

1 $$Precision = \frac{TP}{{TP + FP}}$$

Recall denotes the proportion of actual positive cases correctly identified among all actual positive instances. It is computed using Formula (2).

2 $$Recall = \frac{TP}{{TP + FN}}$$

mAP (mean Average Precision) represents the average AP value across the all categories (c = 11) examined in this study. It is computed using Formula (3).

3 $$mAP = \frac{{\mathop \sum \nolimits_{c = 1}^{C} AP\left( c \right)}}{C}$$

In this calculation, C represents the number of classes being analyzed. Additionally, TP (True Positives), FP (False Positives), and FN (False Negatives) are defined as follows:

- If the IoU between the predicted and actual bounding boxes is greater than 0.5, the detection is classified as a True Positive (TP).
- If the IoU is less than 0.5, it is classified as a False Positive (FP).
- If an actual bounding box is not detected by the model, it is considered a False Negative (FN).

The training process of the YOLO models were conducted using the services of Kaggle, which provides a virtual environment with a Graphics Processing Unit (GPU P100).

## Results and discussion

### Models’ detection performance

**Fig. 4:**
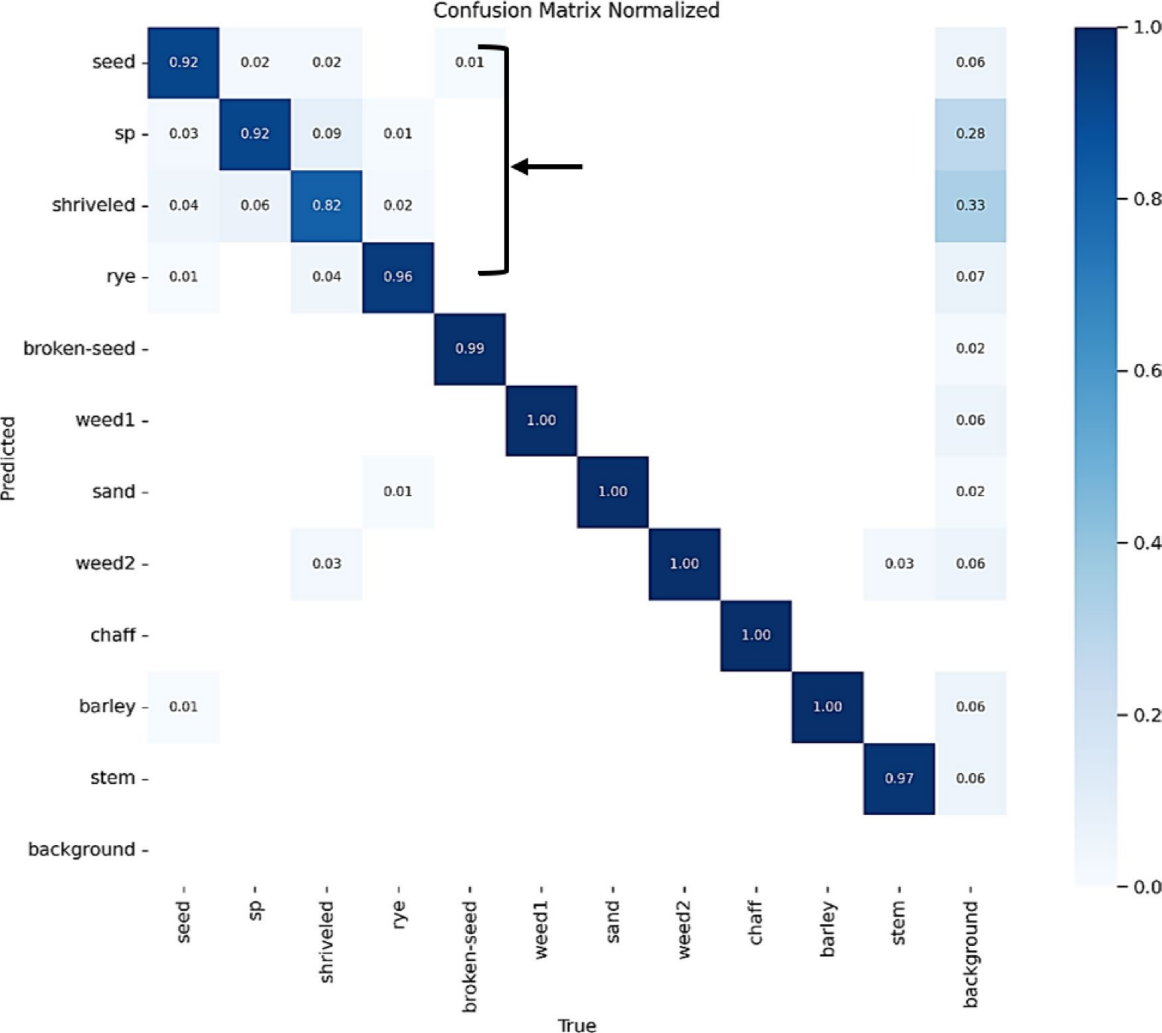
Confusion matrix for YOLOv8x model in wheat impurity detection.

The confusion matrix presented in Fig. 4 illustrates the performance of the YOLOv8x model with an input image size of 640 × 640 in assessing wheat impurities. The matrix demonstrates the model’s effectiveness in accurately identifying most foreign impurities. As shown in the Fig. 4, the model successfully detected impurities such as stones, chaff, weeds, and stems with high accuracy. However, the highest misclassification rates were observed in calsses such as sun pest-damaged wheat grains, rye seeds, shriveled wheat grains, and barley grains. This misclassification is primarily attributed to the strong visual similarities between these classes and regular wheat grains, which makes it challenging for the model to differentiate subtle morphological differences. In addition to visual resemblance, other factors may have contributed to the confusion, such as intra-class variation caused by natural diversity in grain appearance, inconsistencies in image quality (e.g., lighting and resolution), and differences in object size or position within the captured images. For the other examined models, including YOLOv5x, YOLOv5n, and YOLOv8n, the results were comparable to those obtained with YOLOv8x. Notably, classes such as stems, barley, chaff, green weeds (weed2), wild oats (weed1), stones, and broken wheat grains were detected with an accuracy exceeding 98%.

**Fig. 5:**
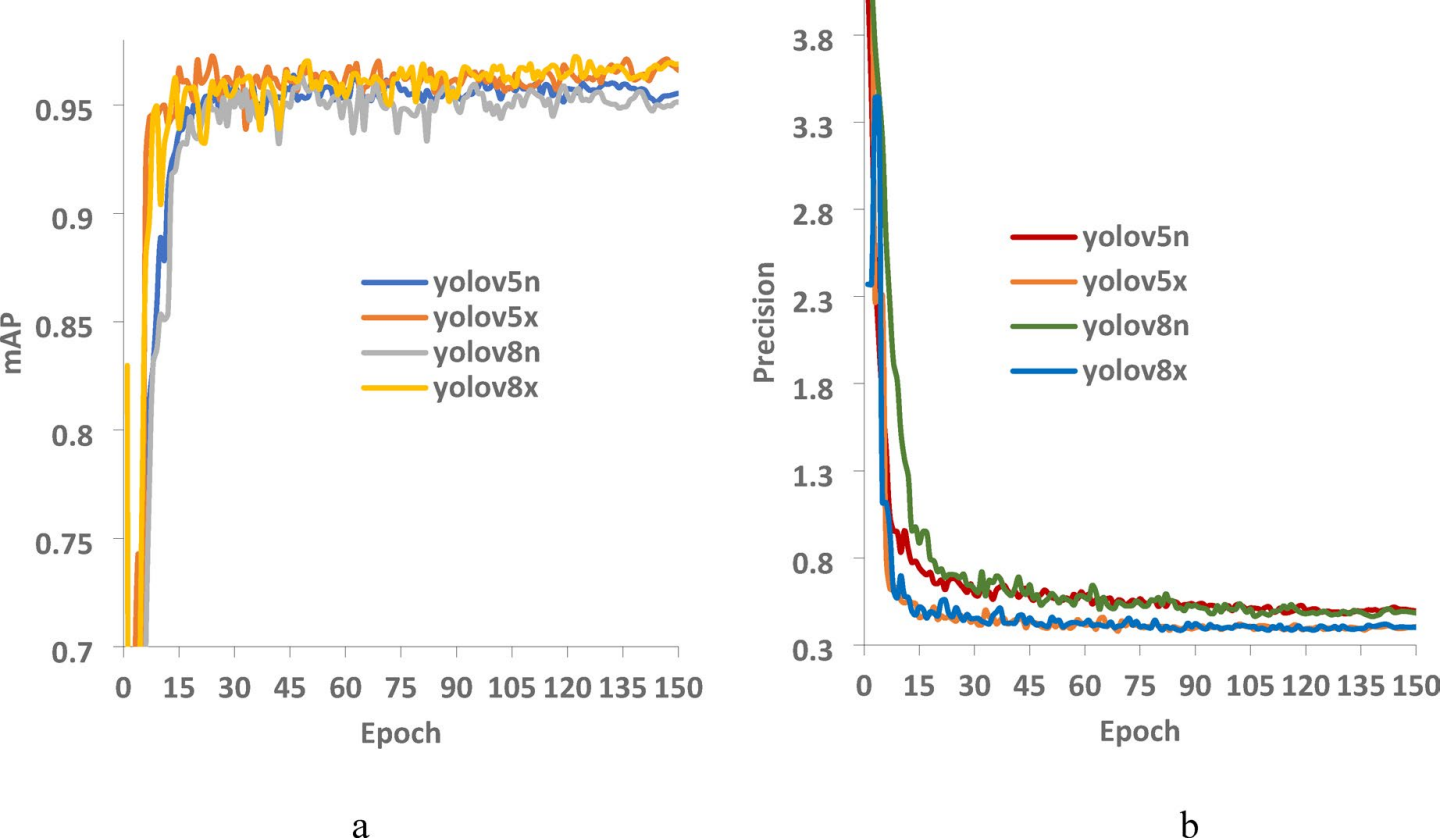
(**a**) The mAP@0.50 and (**b**) the loss values of the evaluated YOLO models during the training process.

Figure 5a illustrates the mAP metric trends for each model throughout the training process. For YOLOv5x and YOLOv8x, the mAP stabilized at 97% after 90 epochs, while for YOLOv5n and YOLOv8n, the mAP converged to 95% after 60 epochs, with no significant improvements in subsequent iterations. The difference in convergence epochs can be attributed to variations in model size and the number of learnable parameters. YOLOv5x and YOLOv8x contain significantly more learnable parameters than YOLOv5n and YOLOv8n, leading to a longer training period before stabilization. The behavior and convergence of both mAP and loss (Fig. 8-b) toward constant values indicate that the training process is complete. Both YOLOv5x and YOLOv8x show decreasing loss curves within 150 epochs before stabilizing. Furthermore, YOLOv5x and YOLOv8x demonstrate faster convergence compared to YOLOv5n and YOLOv8n, suggesting that they extract features more effectively, thereby accelerating the model’s convergence speed.

**Fig. 6:**
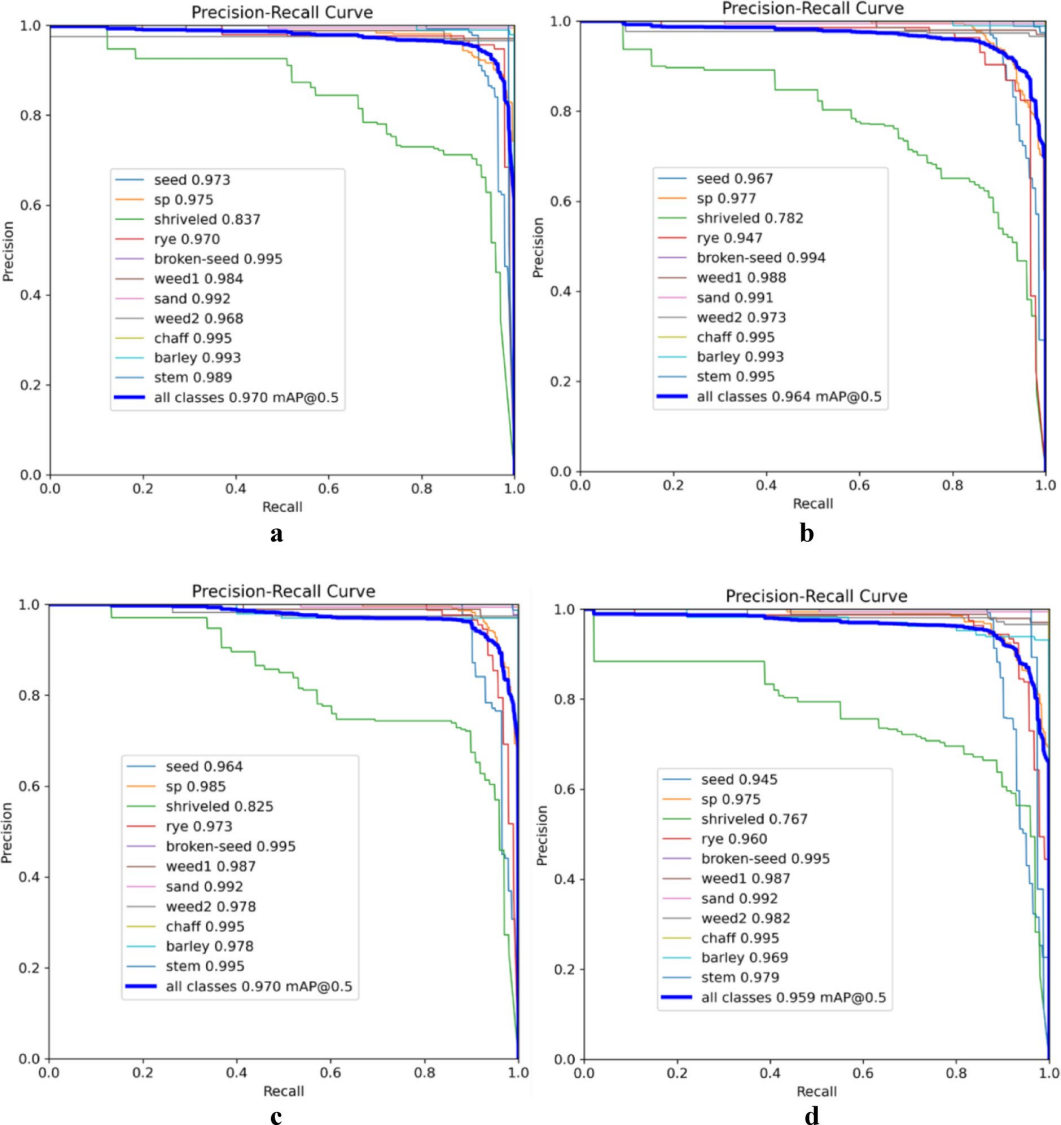
Precision-Recall curves for different models: (**a**) YOLOv5x, (**b**) YOLOv5n, (**c**) YOLOv8x, and (**d**) YOLOv8n for all classes of wheat impurities.

The precision-recall curves of four models—YOLOv5x, YOLOv8x, YOLOv5n, and YOLOv8n—is shown in Fig. 6. This diagram serves as a valuable tool for assessing model performance, where curves positioned closer to the upper-right corner of the plot indicate superior recall and precision. Analysis of these curves demonstrates that most impurities were detected with high accuracy across all models, except for shriveled grains, which posed a greater challenge for precise detection. A comparison of the mAP values reveals that YOLOv5x and YOLOv8x achieved higher overall detection accuracy than their smaller counterparts, YOLOv5n and YOLOv8n. However, despite this performance difference, the relatively small gap in mAP between the larger models and the nano versions suggests that YOLOv5n and YOLOv8n remain viable options for real-time applications where detection speed is a priority. This trade-off between accuracy and processing speed underscores the potential of nano models for deployment in resource-constrained environments or applications requiring rapid detection.

### Effect of image size on the model’s performance

**Fig. 7:**
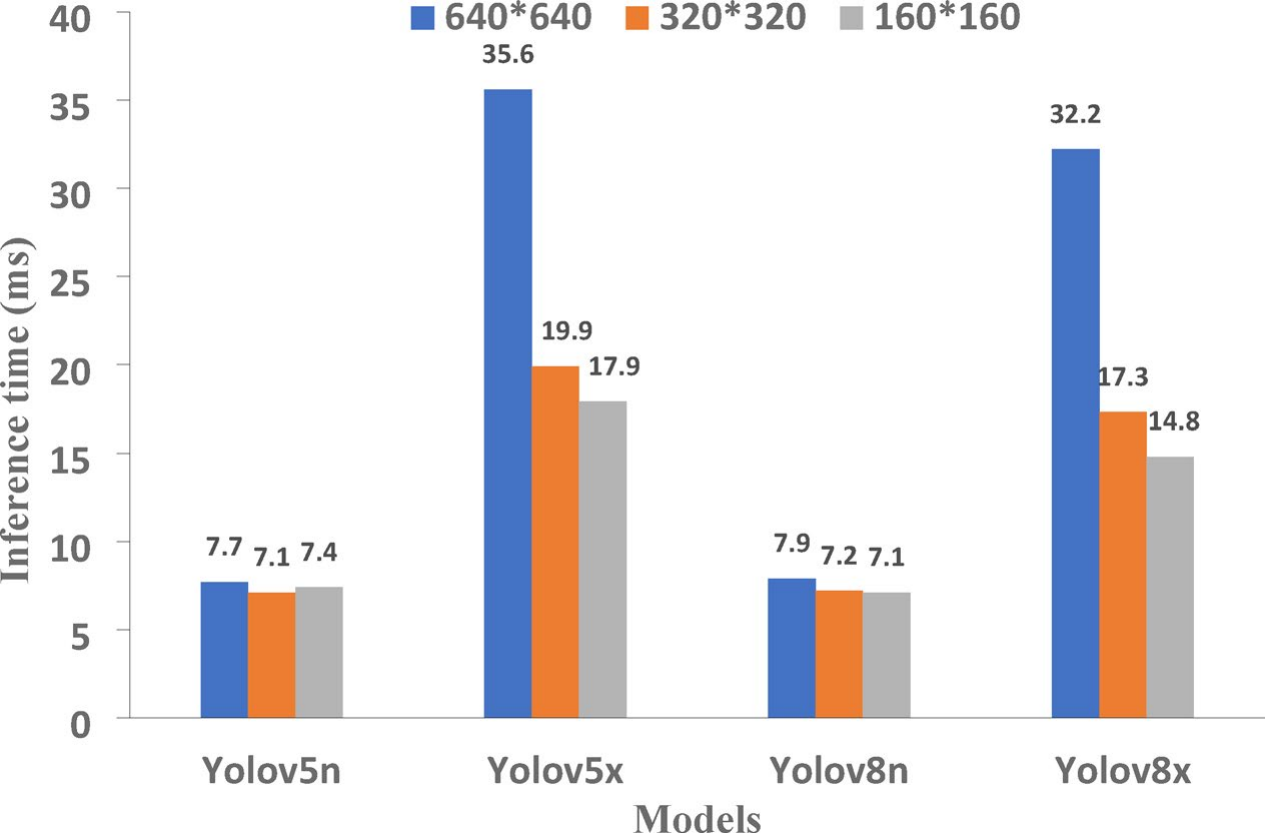
Comparison the inference time (ms) of four models.

In this study, four YOLO models—YOLOv5x, YOLOv8x, YOLOv5n, and YOLOv8n—were evaluated for detecting wheat impurities at three input image resolutions: 640 × 640, 320 × 320, and 160 × 160 pixels. Performance metrics including precision, recall, F1-score, mAP@0.5, and inference time were compared to select the most suitable models. As Table 2 and Fig. 7 show, for applications where real-time detection is essential, the YOLOv5n and YOLOv8n models demonstrate the best performance due to their significantly faster inference times and compact model sizes. At 640 × 640 resolution, YOLOv5n achieves an inference time of 7.7 ms with a mAP of 0.964 and F1-score of 0.948, making it a highly efficient choice for speed-critical tasks. Similarly, YOLOv8n provides comparable performance with an inference time of 7.9 ms and a mAP of 0.960 and F1-score of 0.944. Reducing the input image size to 320 × 320 further improves inference speed for both models, with YOLOv5n reaching 7.1 ms while maintaining an acceptable mAP of 0.954.

**Table 2:** Detection results of impurities by different object detection algorithms at different image size.

Image size	Model	Precision	Recall	mAP@50	F1-score	Inference Time (ms)	Parameters (M)
640*640	yolo5vn	0.946	0.95	0.964	0.948	7.7	2.6
	yolo5vx	0.946	0.965	0.97	0.955	35.6	97.2
	yolo8vn	0.931	0.957	0.96	0.944	7.9	3.2
	yolo8vx	0.953	0.952	0.97	0.952	32.2	68.2
320*320	yolo5vn	0.941	0.936	0.954	0.938	7.1	2.6
	yolo5vx	0.944	0.951	0.959	0.947	19.9	97.2
	yolo8vn	0.938	0.931	0.955	0.934	6.9	3.2
	yolo8vx	0.929	0.939	0.956	0.934	17.3	68.2
160*160	yolo5vn	0.851	0.859	0.906	0.855	7.4	2.6
	yolo5vx	0.897	0.929	0.946	0.913	17.9	97.2
	yolo8vn	0.852	0.906	0.918	0.878	7.1	3.2
	yolo8vx	0.882	0.917	0.935	0.899	14.8	68.2

For applications where detection accuracy and F1-score are critical than speed of detection, the larger models—YOLOv5x and YOLOv8x—outperform their smaller counterparts. At 640 × 640 resolution, YOLOv5x and YOLOv8x both achieve the highest mAP values of 0.970, with F1-scores of 0.955 and 0.952, respectively. Although their inference times are longer (35.6 ms for YOLOv5x and 32.2 ms for YOLOv8x), the accuracy gained justifies their use for applications that accuracy is more important than inference speed or detection time. At 320 × 320 resolution, both models maintain strong performance, with YOLOv5x achieving a mAP of 0.959 and an F1-score of 0.947 in 19.9 ms. This resolution offers a reasonable trade-off between speed and accuracy for tasks where precision remains important but computational time must be reduced.

At 160 × 160 resolution, all models experience a noticeable decline in performance compared to the higher resolutions of 640 × 640 and 320 × 320. The mAP and F1-score values drop significantly, reflecting the reduced accuracy in detecting wheat impurities at this lower resolution. This decline is expected, as the reduced image size results in a loss of detail and features crucial for accurate object detection.

Considering the mAP values and inference time, the results at a resolution of 640 × 640 indicate that increasing the model size leads to only a marginal improvement in accuracy. However, this improvement is not significant enough to justify the substantial reduction in processing speed. Therefore, based on the findings, the YOLOv5n model at a resolution of 640 × 640 emerges as the most optimal choice. Replacing YOLOv5n with a larger model results in only a 0.06% increase in mAP, while inference speed decreases by 362%.

### Assessment of evaluation metrics

As shown in Table 3, both the mAP@50 and the average F1-scores for all impurities, except shriveled grains, are high, demonstrating the strong capability of the YOLOv5n network in recognizing these impurities. This suggests that shriveled grains are more challenging to distinguish due to their less distinct features compared to other impurities. The low recall values for wheat grains, sun pest damaged, and rye further highlight the similarity in patterns among these classes, making their differentiation more challenging, particularly in tasks that demand high performance.

**Table 3:** Performance of the YOLOv5n model in identifying impurities in the 70 images of test dataset.

Class	Precision	Recall	mAP@50	F1-score
all	0.946	0.950	0.964	0.948
Seed	0.992	0.88	0.967	0.933
Sun pest damaged	0.936	0.906	0.977	0.921
Shriveled	0.629	0.867	0.782	0.729
Rye	0.963	0.855	0.947	0.906
broken-seed	0.988	0.977	0.994	0.982
Weed 1	0.968	1	0.988	0.984
sand	0.991	1	0.991	0.995
Weed 2	0.959	1	0.973	0.979
Chaff	0.994	1	0.995	0.997
Barley	0.990	0.994	0.993	0.992
Stem	0.998	0.974	0.995	0.986

In reviewing and comparing the results of this study with previous research, despite significant differences in the models and imaging methods, the outcomes were nearly identical. In a similar study aimed at detecting six types of wheat impurities using terahertz spectral imaging and convolutional neural networks, the average F1-score was reported as 97.83%, which is almost consistent with the present study’s results^3^. However, they only examined six types of impurities, whereas this study investigated ten types of impurities in wheat. Additionally, Qi et al.^27^ utilized the DeepLab-EDA model to detect wheat impurities and broken wheat grains, achieving notable performance with a mean precision of 95.97% and mean recall of 94.83%. Their laboratory detection errors were reported as 7.54% and 6.30%, while field tests yielded error rates of 13.32% and 9.77% for wheat broken rate and impurity rate, respectively. Although their error rates were higher than those observed in our study, it is essential to highlight that their tests were conducted under real field conditions. Additionally, their study focused exclusively on straw as the impurity, thereby excluding other common impurities typically present in wheat samples. A key factor contributing to the superior performance of our study is the sample preparation process; unlike Qi et al.^27^ whose samples were not fully separated, we ensured complete separation of all objects, significantly improving the accuracy of our results.

**Fig. 8:**
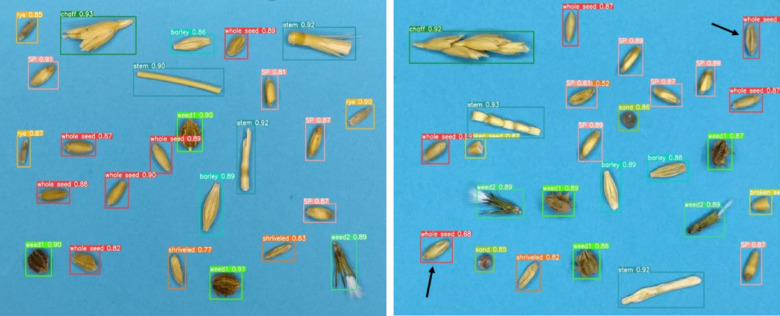
Detection results of the trained YOLOv5n model for identifying wheat impurities.

Figure 8 illustrates the output of the YOLOv5n model in detecting wheat impurities within the test image. As observed, the model accurately identified all impurities, including wheat grains. However, a misclassification occurred in two instances, where shriveled grains were incorrectly recognized as whole grains.

## Conclusion

This study demonstrates the potential of deep learning–based systems for real-time detection of wheat impurities. Such approaches could be applied to impurity monitoring in storage silos, seed quality assessment, and a range of broader agronomic practices. The results revealed that employing extra-large models such as YOLOv5x and YOLOv8x did not significantly enhance impurity detection accuracy but notably increased image processing time. For applications such as impurity detection at wheat storage silos, where detection speed is not a priority, using YOLOv5x or YOLOv8x may be preferable due to their slight improvement in accuracy. However, for real-time applications where both speed and accuracy are critical, the YOLOv5n model proves to be a more suitable choice. A general review of the results showed that the models faced the greatest challenge in detecting shriveled and sun pest grains. Since robust pattern recognition requires a large dataset, expanding the number of images for shriveled and sun pest damaged grains is recommended to enhance network performance. Future work will emphasize testing and validating the developed model under real operating conditions. Particular attention will be given to evaluating its performance in grain silos and commercial seed quality assessment centers. Additional studies will also consider robustness against variations in lighting, grain variety, and impurity types, as well as integration with automated sorting systems for large-scale applications.

## Data Availability

The data for this manuscript are not publicly available but may be accessed upon request to the corresponding author.
